# A Study of the Prediction of Mortality in a Tertiary Care Hospital Using the Modified Sick Neonatal Score (MSNS): An Observational Cross-Sectional Study

**DOI:** 10.7759/cureus.38484

**Published:** 2023-05-03

**Authors:** Prakash Reddy, Beere Gowda, Abhinay R

**Affiliations:** 1 Pediatrics, Sri Devaraj Urs Medical College, Kolar, IND

**Keywords:** neonatal intensive care unit (nicu), neonatal care, neonatal mortality, modified sick neonatal score, development, death, sick neonatal score, neonates, intensive care unit

## Abstract

Purpose: India is a major contributor to neonatal deaths worldwide. There is a paucity of amenities for the management of neonatal health issues in rural areas of our country. Hence, there is a need to invent a reliable scoring system for the analysis of neonatal mortality.

Aim: The aim of the study is to evaluate the Modified Sick Neonatal Score (MSNS) as a predictor of mortality in neonatal care units in resource-limited settings.

Materials and methods: This cross-sectional observational study was performed in the intensive care unit of our hospital. All the data were collected and analyzed using IBM Corp.'s Statistical Package for Social Sciences (SPSS) software.

Results: Overall, 71 participants were considered for the present study. The common clinical diagnoses noticed in our participants were meconium aspiration, malformation, and jaundice. The MSNS score compared between expired and discharged participants is found to be statistically significant with p<0.05.

Conclusion: The MSNS scoring system is considered an ideal scoring system for detecting early mortality in neonates.

## Introduction

Despite advancements in health services for neonates, the risk during the neonatal phase persists, as seen in the increasing mortality rates. The majority of neonatal deaths occur on the first day and during the first week of neonatal life; this number accounts for two-thirds of neonatal deaths [1]. If proper control measures are not implemented, it would be difficult to reach the goal of reducing neonatal mortality by targeting 12 per 1,000 live births by 2030 in accordance with the Sustainable Development Goals [2]. According to the National Family Health Survey (NFHS-5), the neonatal mortality rate in India is 24.9 per 1,000 live births. A scarcity of effective treatment can cause high mortality rates, which demands a proper scoring system that would enable simple, reliable, and reproducible evaluation with the possibility of making standardised assessments concerning the performances of various stakeholders. Such a scoring system will facilitate comparisons of well-being among neonates across various instances [3-5].

In recent times, several scoring systems have been invented, such as the Score for Neonatal Acute Physiology (SNAP), SNAP-II, Score for Neonatal Acute Physiology-Perinatal Extension (SNAPPE), SNAPPE-II, and the Neonatal Therapeutic Intervention Scoring System (NTISS). However, all of these systems had certain limitations [6,7]. The Sick Neonatal Score (SNS) and Extended Sick Neonatal Score (ESNS) systems were implemented but faced accessibility and availability issues [8, 9]. Hence, these systems were modified by adding other parameters such as birth weight and gestational age; such modified systems are referred to as the Modified Sick Neonatal Score (MSNS) [10-14].

Given the above context, this study aimed to examine the prediction of mortality using MSNS in a tertiary care hospital in order to help evaluate the efficacy of the MSNS system. Establishing the efficacy of such an easy-to-perform neonatal scoring method is important for improving neonatal disease outcomes, upgrading the performance of Special Newborn Care Units (SNCUs), and facilitating early referral of sick neonates to better-equipped centres.

## Materials and methods

This cross-sectional observational study was performed in the Department of Paediatrics of Sri Devaraj Urs Medical College, a tertiary care hospital in Kolar, India. The study was approved by the institutional ethical committee and was conducted from October to December 2022 (approval number: DMC/KLR/IEC/554/2022-23). Consent was obtained by the parents of all the neonates who were included and informed about the aim of the study. The sample size for this study was calculated by predicting the sensitivity and specificity of MSNS < 10 with an absolute precision of 1% and a 98% confidence interval using the formula N = Z21−αp (1-p)/d2, where N = number of samples, α = level of significance, Z1-α= corresponding normal standard variant, P = sensitivity (%), and d = absolute precision. The sample size was found to be 71.

Inclusion Criteria

Neonates admitted to the NICU were included in the present study.

Exclusion Criteria

Parents who did not give consent for the participation of their neonates in the study, neonates on ventilator support at admission, and neonates discharged against medical advice were excluded from the study.

A semi-structured proforma was used to record demographic details, gestational age, birth weight, important clinical findings with investigations, and diagnosis. A glucometer was used to check random blood sugar, and oxygen was supplied on transport. All babies were followed up until discharge, and the outcome was noted. Disease severity was assessed immediately at admission and scored using the MSNS. The MSNS score determination has eight parameters, of which six were adapted from the sick neonatal score (SNS) score. The details of MSNS parameters are mentioned in Table 1.

**Table 1:** Parameters of the modified sick neonatal score (MSNS)

Parameter	Score 0	Score 1	Score 2
Respiratory effort	Apnea or grunt	Tachypnea (respiratory rate >60/min) with or without retractions	Normal (respiratory rate 40–60/min)
Heart rate	Bradycardia or asystole	Tachycardia (>160/min)	Normal (100–160/min)
Axillary temperature (°C)	<36	36–36.5	36.5–37.5
Capillary refilling time (s)	>5	3–5	<3
Random blood sugar (mg/dl)	<40	40–60	>60
SpO_2_ (in room air)	<85	85–92	>92
Gestational age (in weeks)	<32 weeks	32 to 36 weeks + 6/7 days	37 weeks and above
Birth weight (kg)	<1.5	1.5–2.49	2.5 or above
Total	Maximum 16		

All the data were collected, coded, and analysed using IBM Corp.'s Statistical Package for Social Sciences (SPSS) software. Results were manifested as a mean with standard deviation, a median with interquartile ranges, and percentages using appropriate tables. The Chi-square test was used to depict the association between the individual parameters and the outcome. The receiver operating characteristic (ROC) curve was brought into being with MSNS as the test variable to predict mortality. The optimum cutoff value was obtained from the ROC curve. Sensitivity and specificity were calculated for the cutoff score.

## Results

A total of 71 neonates were considered for the present study. The MSNS, or mean (standard deviation) score, in expired individuals, was 9.11 and in discharged individuals was 12.9. There was a statistically significant difference between expired and discharged individuals (p<0.001). The MSNS score was significantly lower in expired individuals compared to discharged individuals (Table 2).

**Table 2:** Distribution of parameters based on the modified sick neonatal score

Parameters	Score	Frequency	Discharged	Expired	p-value
Respiratory effort	0 1 2	12 9 50	0 31 31	0 5 4	<0.002
Heart rate	0 1 2	5 9 57	0 31 31	0 4 5	<0.002
Axillary temperature	0 1 2	2 34 34	0 31 31	0 4 5	<0.002
Capillary refilling time	0 1 2	3 5 63	0 31 31	0 4 5	<0.002
Random blood sugar	0 1 2	01 13 57	0 31 31	0 4 5	<0.002
SpO2	0 1 2	5 5 61	0 31 31	0 4 5	<0.002
Gestational age	0 1 2	04 20 47	0 31 31	0 4 5	<0.002
Birth weight	0 1 2	06 22 43	0 31 31	0 4 5	<0.002
Total Score					<0.002

The Mann-Whitney test exhibits the difference between scores of discharged and expired (Table 3), which were statistically significant except for SpO2 and birthweight.

**Table 3:** Median among the parameters IQR: interquartile range

MSNS parameter	Outcome	Median (IQR)	Mann-Whitney test (Z)	p-value
Respiratory effort	Discharged/ Expired	1/ 1	5.2	<0.001
Heart rate	Discharged/ Expired	2/ 1	9.8	<0.001
Axillary temperature	Discharged/ Expired	1/ 0	10.9	<0.001
Capillary refilling time	Discharged/ Expired	2/ 0	6.2	<0.001
Random blood sugar	Discharged/ Expired	2/ 2	7.9	<0.001
SpO2	Discharged/ Expired	2/ 1	2.3	0.005
Gestational age	Discharged/ Expired	2/ 1	6.2	<0.001
Birth weight	Discharged/ Expired	1/ 1	1.3	0.12

**Figure 1:**
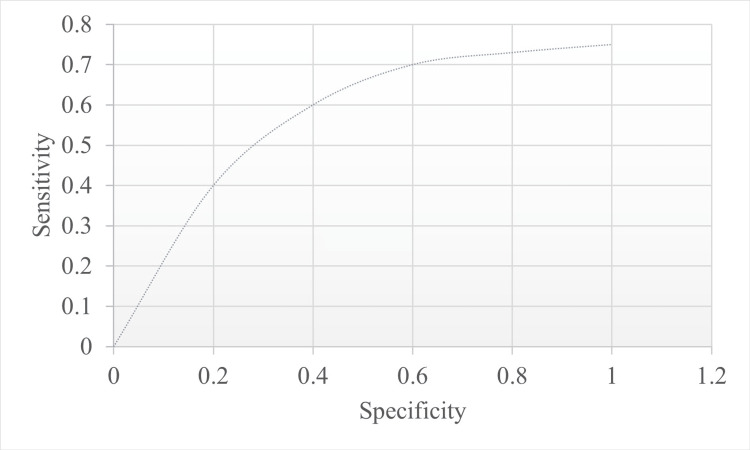
ROC curve with the total modified sick neonatal score

In the present study, MSNS had a sensitivity of 69%, a specificity of 87.3%, and an optimum cutoff score ≤ 9 to predict mortality. The ROC curve was 0.88 (Figure 1).

**Figure 2:**
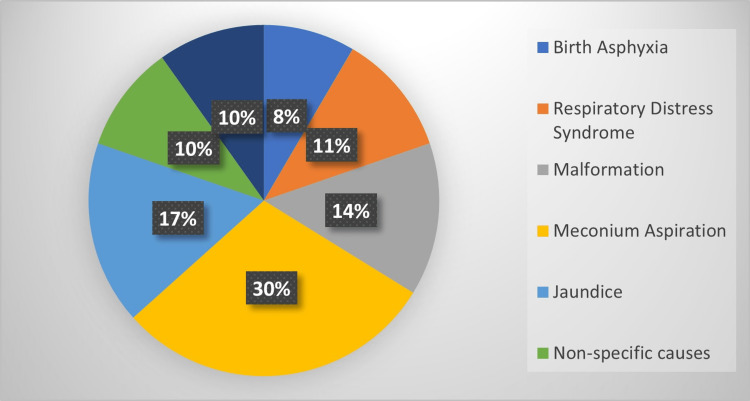
Clinical diagnosis of participants

The clinical diagnostic parameters noticed among the study participants were birth asphyxia, respiratory distress, malformation, meconium aspiration syndrome, jaundice, nonspecific causes, and sepsis (Figure 2).

The baseline characteristics of the participants are mentioned in Table 4. Males represented 83%, and females represented 16% of the sample. Most of them were term babies, representing 80%, and the least of them were preterm babies. The majority of neonates born were delivered by vaginal delivery rather than caesarean and fell under primi gravida. Of the total number of neonates included in the study, 87% were discharged, and 12% expired.

**Table 4:** Baseline characteristics of the neonates

Characteristics	Frequency (%)
Gender	
Male	59 (83%)
Female	12 (16%)
Gestational age	
Preterm (36.6 weeks and less)	14 (19%)
Term (37 weeks to 42 weeks)	57 (80%)
Mode of delivery	
Vaginal	55 (77%)
Caesarean	16 (22%)
Parity	
Primi	58 (81%)
Multi	13 (18%)
Outcome	
Discharged	62 (87%)
Expired	9 (12%)

## Discussion

The MSNS system is easy to implement, less time-consuming, applicable in the early course of hospitalisation, and reproducible for assessing mortality and morbidity among all neonatal groups. Compared to the MSNS system, the other novel devices invented so far are not easily available everywhere. Also, the MSNS system is preferred in limited-resource settings [15-17]. Hence, in the present study, disease severity in neonates was assessed, which is an essential tool for minimising complications. In the present study, the MSNS parameters of 71 neonates admitted to the intensive care unit of our hospital were assessed.

In the present study, the mean MSNS was lower in expired neonates (10) than in those who were discharged (14). These results were similar to other studies, wherein a very low MSNS was noticed in expired neonates compared to discharged neonates, whereas MSNS had better sensitivity and specificity scores than sick neonatal score (SNS) and extended sick neonatal score (ESNS). In the current study, 80% of the neonates were born at term, 19% were preterm, low birth weight was noted in 30%, and 60% weighed more than 2500 grams. The clinical findings of our study participants were similar to those in other studies where birth asphyxia, respiratory distress, and sepsis remained common clinical problems (Figure 2). We noticed a greater number of males among our participants, which may be attributed to biological vulnerability and several other social causes, such as a preference for male children. The mortality rate observed in the present study might be due to complications observed in the mother, which are similar to the findings of studies by Verma et al., Chheda et al., and Shah et al. [18-20].

In summary, in the present study, compared to other scoring systems, MSNS could be applied to all participants easily during hospitalisation and could be used for both term and preterm babies. Hence, it serves as a better-suited neonatal disease severity score for SNCUs, given their limited resources. MSNS had a sensitivity of 67% and a specificity of 87 when an optimum cutoff score of <10 was used to predict mortality. These results were compared to other studies where the ROC curve value was less than 10 with similar specific and sensitive values [10, 21, 22].

As per a report by the Ministry of Health and Family Welfare, Government of India, there were 525 Special Newborn Care Units (SNCUs) all over India. And 75% of babies admitted to the hospital belong to the underprivileged section. Such units are established at district hospitals and sub-district hospitals with an annual delivery load of more than 3000, and the mortality rate was found to be 2% to 19%. There is a necessity to compare the performance of various SNCUs to initiate corrective measures in those units with poor performance. The comparison of mortality between the various units would be more meaningful if it were adjusted for the disease severity scores of the neonates admitted. Hence, MSNS scores developed recently helped in assessing the severity and complications in neonates [15].

Despite the various advantages of the MSNS system, there exist certain limitations within the system in certain instances, such as the adequacy of antenatal care, chorioamnionitis, maternal diabetes, events during labour and delivery, and hypertension. These presenting factors impact the MSNS system negatively. Apart from these instances, there are several other limiting factors, such as nosocomial infections. The scoring was performed only once at admission, and serial scoring may have provided additional information [7].

The present study is a single-centre study and therefore requires extensive validation before implementation. Also, several other studies are needed to confirm applicability across various settings.

## Conclusions

The MSNS is a useful neonatal disease severity score, as per the results of the study. It has better sensitivity and specificity, which are noted in the study observations. It is easy to use, even in minimal-resource settings in low- and middle-income countries.
